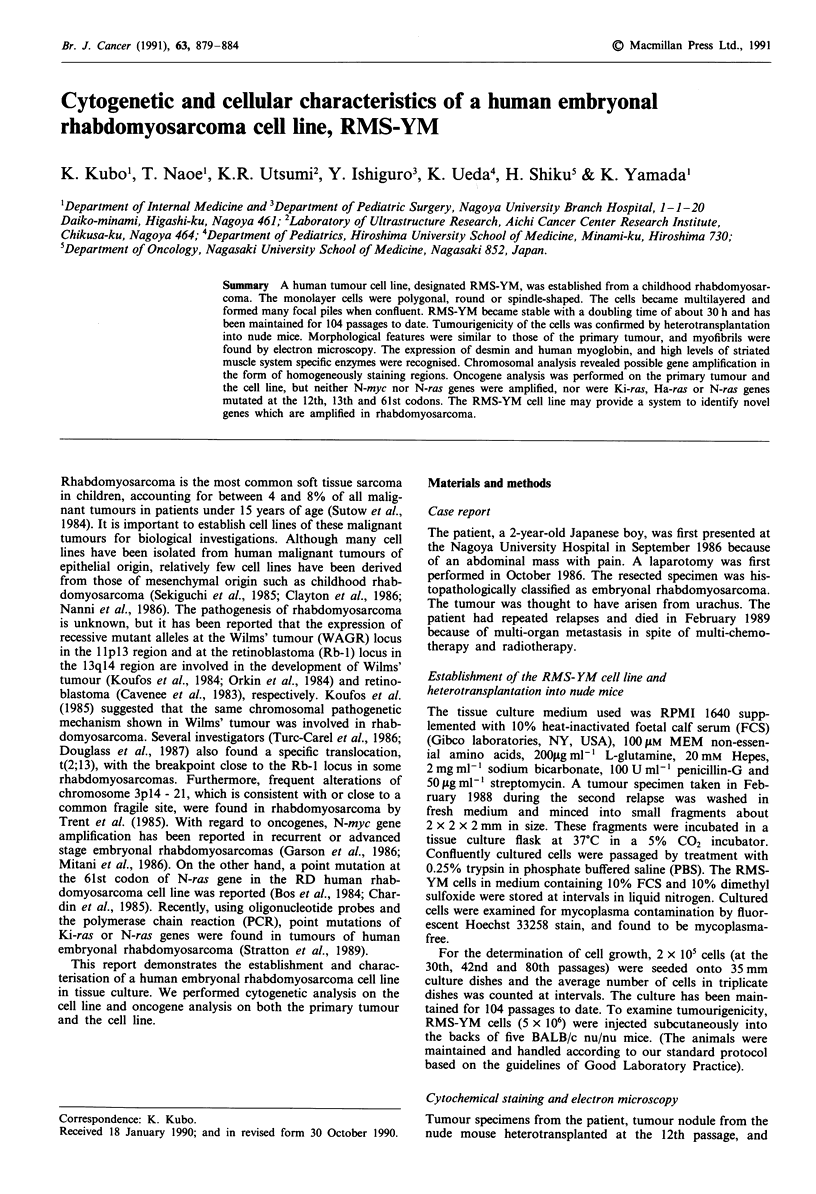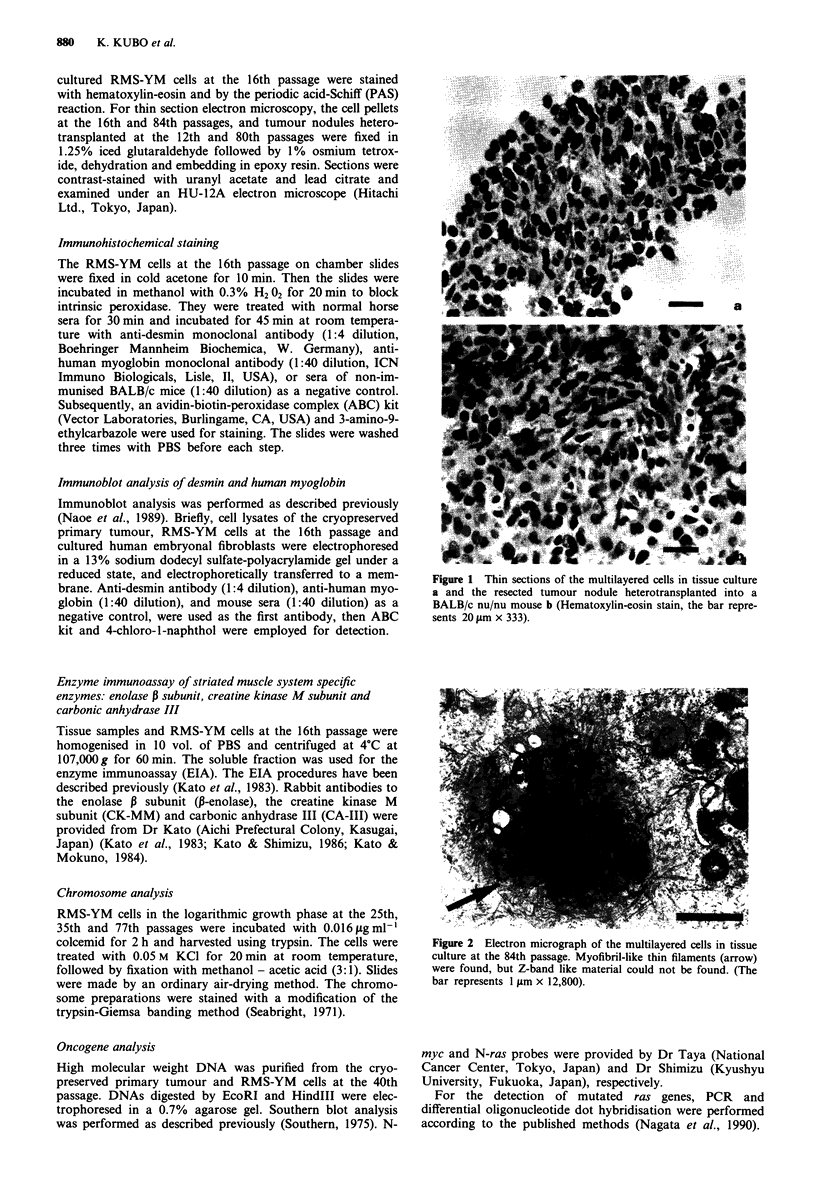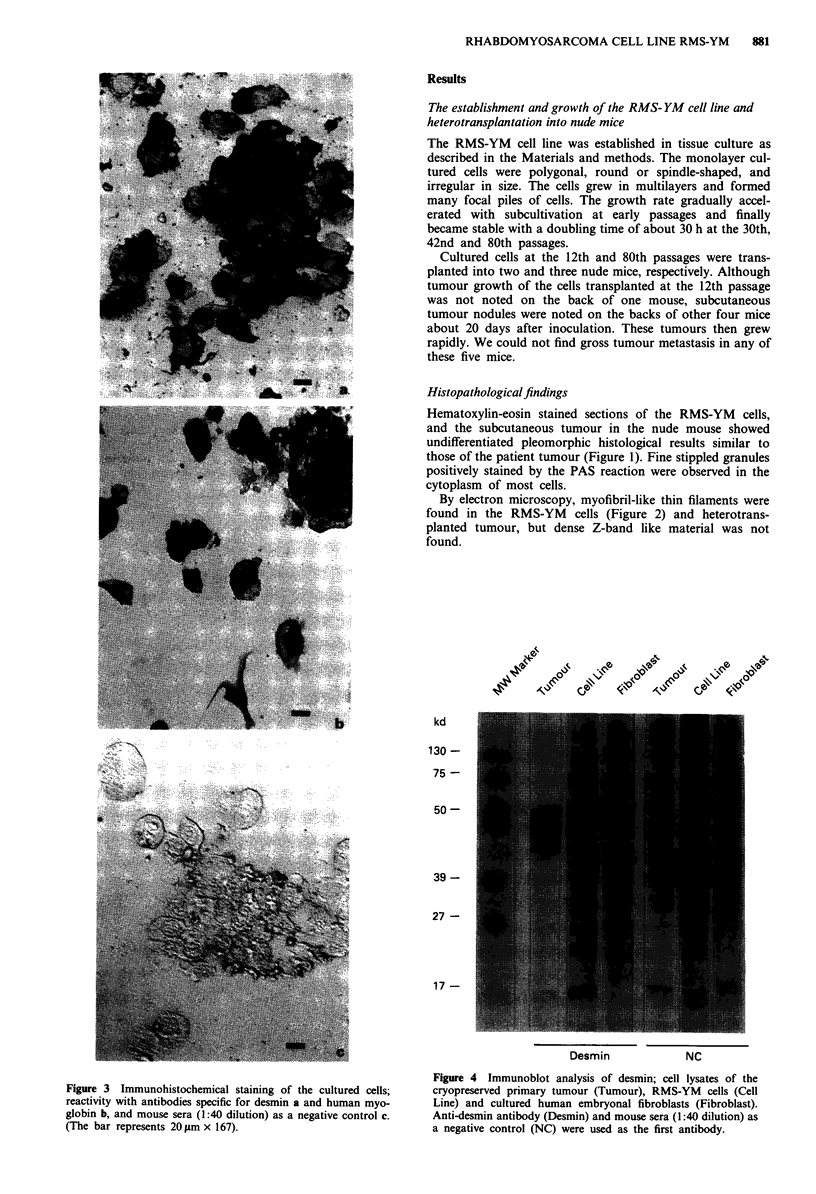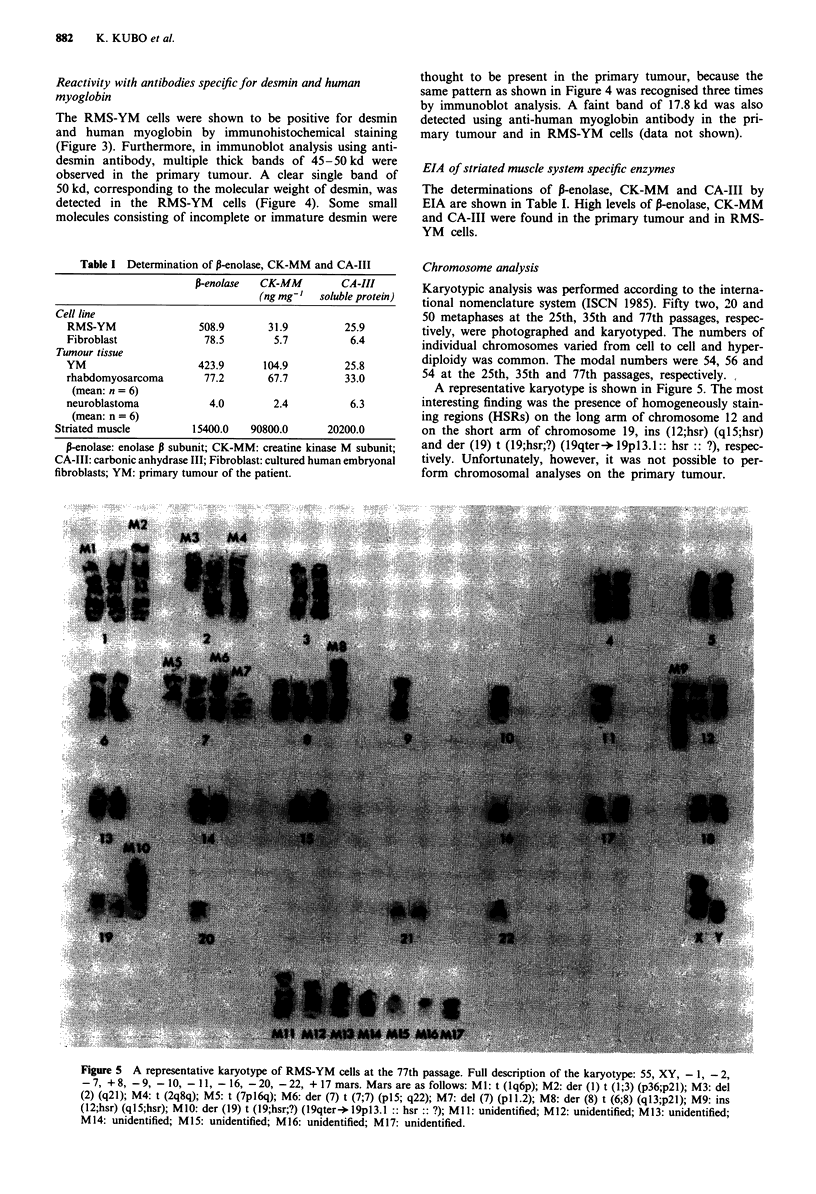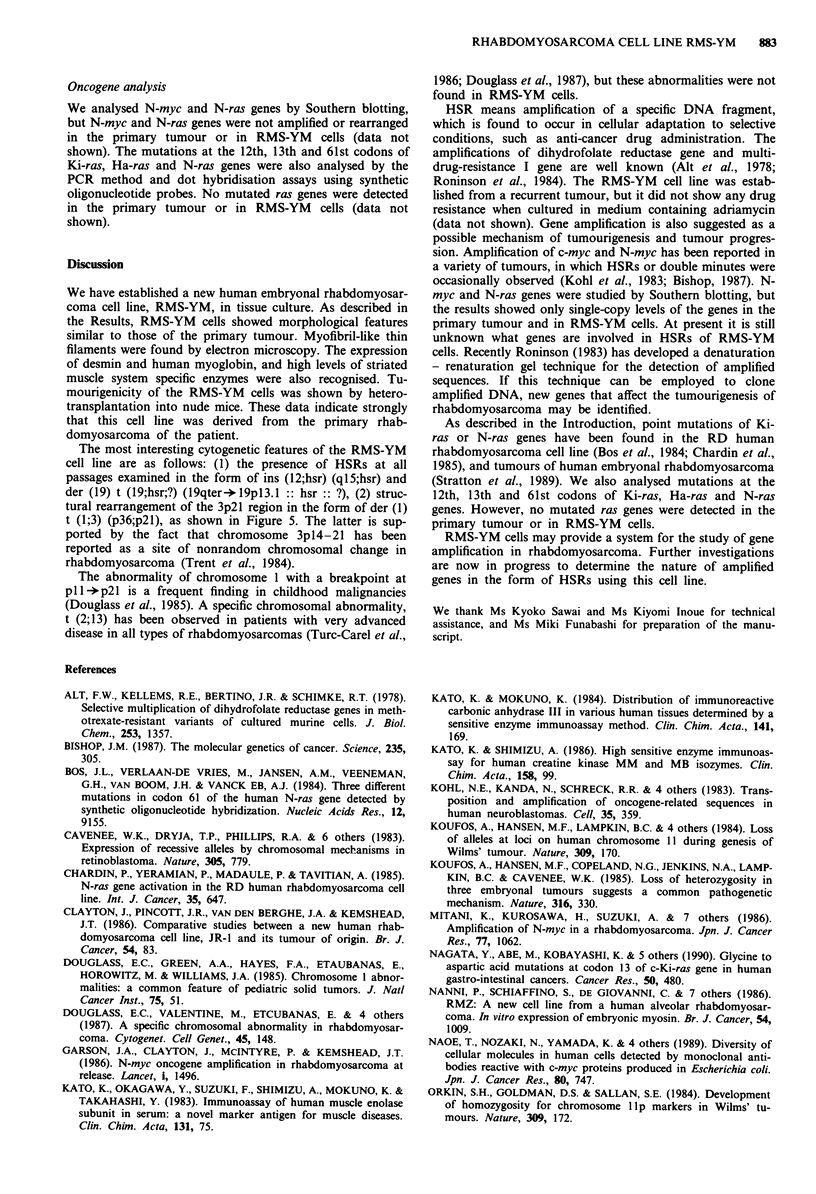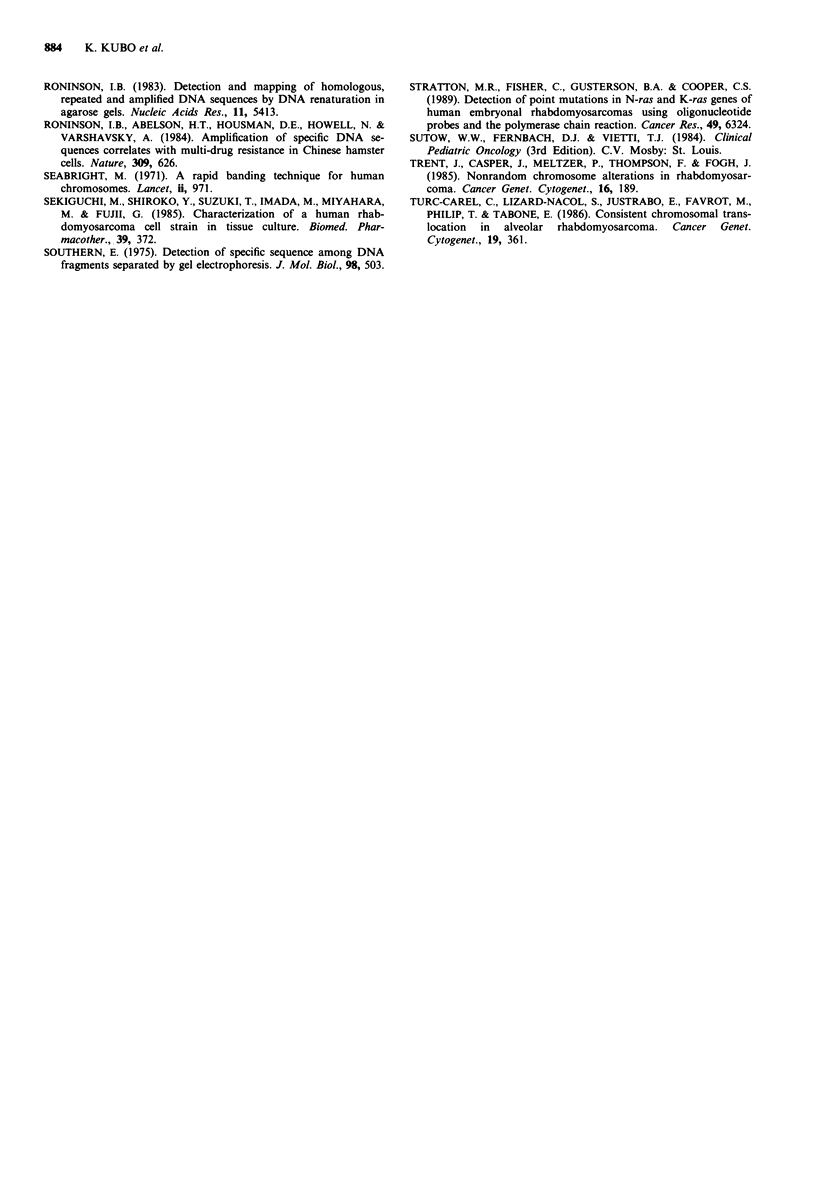# Cytogenetic and cellular characteristics of a human embryonal rhabdomyosarcoma cell line, RMS-YM.

**DOI:** 10.1038/bjc.1991.193

**Published:** 1991-06

**Authors:** K. Kubo, T. Naoe, K. R. Utsumi, Y. Ishiguro, K. Ueda, H. Shiku, K. Yamada

**Affiliations:** Department of Internal Medicine, Nagoya University Branch Hospital, Japan.

## Abstract

**Images:**


Br. J. Cancer (1991), 63, 879–884 © Macmillan Press Ltd., 1991

### Cytogenetic and cellular characteristics of a human embryonal rhabdomyosarcoma cell line, RMS-YM

K. Kubo[1], T. Naoe[1], K.R. Utsumi[2], Y. Ishiguro[3], K. Ueda[4], H. Shiku[5] & K. Yamada[1]

[1]*Department of Internal Medicine and* [3]*Department of Pediatric Surgery, Nagoya University Branch Hospital, 1-1-20 Daiko-minami, Higashi-ku, Nagoya 461;* [2]*Laboratory of Ultrastructure Research, Aichi Cancer Center Research Institute, Chikusa-ku, Nagoya 464;* [4]*Department of Pediatrics, Hiroshima University School of Medicine, Minami-ku, Hiroshima 730;* [5]*Department of Oncology, Nagasaki University School of Medicine, Nagasaki 852, Japan.*

**Summary** A human tumour cell line, designated RMS-YM, was established from a childhood rhabdomyosarcoma. The monolayer cells were polygonal, round or spindle-shaped. The cells became multilayered and formed many focal piles when confluent. RMS-YM became stable with a doubling time of about 30 h and has been maintained for 104 passages to date. Tumourigenicity of the cells was confirmed by heterotransplantation into nude mice. Morphological features were similar to those of the primary tumour, and myofibrils were found by electron microscopy. The expression of desmin and human myoglobin, and high levels of striated muscle system specific enzymes were recognised. Chromosomal analysis revealed possible gene amplification in the form of homogeneously staining regions. Oncogene analysis was performed on the primary tumour and the cell line, but neither N-*myc* nor N-*ras* genes were amplified, nor were Ki-*ras*, Ha-*ras* or N-*ras* genes mutated at the 12th, 13th and 61st codons. The RMS-YM cell line may provide a system to identify novel genes which are amplified in rhabdomyosarcoma.

Rhabdomyosarcoma is the most common soft tissue sarcoma in children, accounting for between 4 and 8% of all malignant tumours in patients under 15 years of age (Sutow *et al.*, 1984). It is important to establish cell lines of these malignant tumours for biological investigations. Although many cell lines have been isolated from human malignant tumours of epithelial origin, relatively few cell lines have been derived from those of mesenchymal origin such as childhood rhabdomyosarcoma (Sekiguchi *et al.*, 1985; Clayton *et al.*, 1986; Nanni *et al.*, 1986). The pathogenesis of rhabdomyosarcoma is unknown, but it has been reported that the expression of recessive mutant alleles at the Wilms' tumour (WAGR) locus in the 11p13 region and at the retinoblastoma (Rb-1) locus in the 13q14 region are involved in the development of Wilms' tumour (Koufos *et al.*, 1984; Orkin *et al.*, 1984) and retinoblastoma (Cavenee *et al.*, 1983), respectively. Koufos *et al.* (1985) suggested that the same chromosomal pathogenetic mechanism shown in Wilms' tumour was involved in rhabdomyosarcoma. Several investigators (Turc-Carel *et al.*, 1986; Douglass *et al.*, 1987) also found a specific translocation, t(2;13), with the breakpoint close to the Rb-1 locus in some rhabdomyosarcomas. Furthermore, frequent alterations of chromosome 3p14 - 21, which is consistent with or close to a common fragile site, were found in rhabdomyosarcoma by Trent *et al.* (1985). With regard to oncogenes, N-*myc* gene amplification has been reported in recurrent or advanced stage embryonal rhabdomyosarcomas (Garson *et al.*, 1986; Mitani *et al.*, 1986). On the other hand, a point mutation at the 61st codon of N-*ras* gene in the RD human rhabdomyosarcoma cell line was reported (Bos *et al.*, 1984; Chardin *et al.*, 1985). Recently, using oligonucleotide probes and the polymerase chain reaction (PCR), point mutations of Ki-*ras* or N-*ras* genes were found in tumours of human embryonal rhabdomyosarcoma (Stratton *et al.*, 1989).

This report demonstrates the establishment and characterisation of a human embryonal rhabdomyosarcoma cell line in tissue culture. We performed cytogenetic analysis on the cell line and oncogene analysis on both the primary tumour and the cell line.

Correspondence: K. Kubo.
Received 18 January 1990; and in revised form 30 October 1990.

### Materials and methods

### *Case report*

The patient, a 2-year-old Japanese boy, was first presented at the Nagoya University Hospital in September 1986 because of an abdominal mass with pain. A laparotomy was first performed in October 1986. The resected specimen was histopathologically classified as embryonal rhabdomyosarcoma. The tumour was thought to have arisen from urachus. The patient had repeated relapses and died in February 1989 because of multi-organ metastasis in spite of multi-chemotherapy and radiotherapy.

### *Establishment of the RMS-YM cell line and heterotransplantation into nude mice*

The tissue culture medium used was RPMI 1640 supplemented with 10% heat-inactivated foetal calf serum (FCS) (Gibco laboratories, NY, USA), 100 $\mu$M MEM non-essenial amino acids, 200 $\mu g\ ml^{-1}$ L-glutamine, 20 mM Hepes, 2 $mg\ ml^{-1}$ sodium bicarbonate, 100 $U\ ml^{-1}$ penicillin-G and 50 $\mu g\ ml^{-1}$ streptomycin. A tumour specimen taken in February 1988 during the second relapse was washed in fresh medium and minced into small fragments about $2 \times 2 \times 2$ mm in size. These fragments were incubated in a tissue culture flask at 37°C in a 5% $CO_2$ incubator. Confluently cultured cells were passaged by treatment with 0.25% trypsin in phosphate buffered saline (PBS). The RMS-YM cells in medium containing 10% FCS and 10% dimethyl sulfoxide were stored at intervals in liquid nitrogen. Cultured cells were examined for mycoplasma contamination by fluorescent Hoechst 33258 stain, and found to be mycoplasma-free.

For the determination of cell growth, $2 \times 10^5$ cells (at the 30th, 42nd and 80th passages) were seeded onto 35 mm culture dishes and the average number of cells in triplicate dishes was counted at intervals. The culture has been maintained for 104 passages to date. To examine tumourigenicity, RMS-YM cells ($5 \times 10^6$) were injected subcutaneously into the backs of five BALB/c nu/nu mice. (The animals were maintained and handled according to our standard protocol based on the guidelines of Good Laboratory Practice).

### *Cytochemical staining and electron microscopy*

Tumour specimens from the patient, tumour nodule from the nude mouse heterotransplanted at the 12th passage, and cultured RMS-YM cells at the 16th passage were stained with hematoxylin-eosin and by the periodic acid-Schiff (PAS) reaction. For thin section electron microscopy, the cell pellets at the 16th and 84th passages, and tumour nodules heterotransplanted at the 12th and 80th passages were fixed in 1.25% iced glutaraldehyde followed by 1% osmium tetroxide, dehydration and embedding in epoxy resin. Sections were contrast-stained with uranyl acetate and lead citrate and examined under an HU-12A electron microscope (Hitachi Ltd., Tokyo, Japan).

*Immunohistochemical staining*

The RMS-YM cells at the 16th passage on chamber slides were fixed in cold acetone for 10 min. Then the slides were incubated in methanol with 0.3% $H_2O_2$ for 20 min to block intrinsic peroxidase. They were treated with normal horse sera for 30 min and incubated for 45 min at room temperature with anti-desmin monoclonal antibody (1:4 dilution, Boehringer Mannheim Biochemica, W. Germany), anti-human myoglobin monoclonal antibody (1:40 dilution, ICN Immuno Biologicals, Lisle, Il, USA), or sera of non-immunised BALB/c mice (1:40 dilution) as a negative control. Subsequently, an avidin-biotin-peroxidase complex (ABC) kit (Vector Laboratories, Burlingame, CA, USA) and 3-amino-9-ethylcarbazole were used for staining. The slides were washed three times with PBS before each step.

*Immunoblot analysis of desmin and human myoglobin*

Immunoblot analysis was performed as described previously (Naoe *et al.*, 1989). Briefly, cell lysates of the cryopreserved primary tumour, RMS-YM cells at the 16th passage and cultured human embryonal fibroblasts were electrophoresed in a 13% sodium dodecyl sulfate-polyacrylamide gel under a reduced state, and electrophoretically transferred to a membrane. Anti-desmin antibody (1:4 dilution), anti-human myoglobin (1:40 dilution), and mouse sera (1:40 dilution) as a negative control, were used as the first antibody, then ABC kit and 4-chloro-1-naphthol were employed for detection.

**Figure 1** Thin sections of the multilayered cells in tissue culture **a** and the resected tumour nodule heterotransplanted into a BALB/c nu/nu mouse **b** (Hematoxylin-eosin stain, the bar represents 20 μm × 333).

*Enzyme immunoassay of striated muscle system specific enzymes: enolase β subunit, creatine kinase M subunit and carbonic anhydrase III*

Tissue samples and RMS-YM cells at the 16th passage were homogenised in 10 vol. of PBS and centrifuged at 4°C at 107,000 ***g*** for 60 min. The soluble fraction was used for the enzyme immunoassay (EIA). The EIA procedures have been described previously (Kato *et al.*, 1983). Rabbit antibodies to the enolase β subunit (β-enolase), the creatine kinase M subunit (CK-MM) and carbonic anhydrase III (CA-III) were provided from Dr Kato (Aichi Prefectural Colony, Kasugai, Japan) (Kato *et al.*, 1983; Kato & Shimizu, 1986; Kato & Mokuno, 1984).

*Chromosome analysis*

RMS-YM cells in the logarithmic growth phase at the 25th, 35th and 77th passages were incubated with 0.016 $\mu g\ ml^{-1}$ colcemid for 2 h and harvested using trypsin. The cells were treated with 0.05 M KCl for 20 min at room temperature, followed by fixation with methanol – acetic acid (3:1). Slides were made by an ordinary air-drying method. The chromosome preparations were stained with a modification of the trypsin-Giemsa banding method (Seabright, 1971).

**Figure 2** Electron micrograph of the multilayered cells in tissue culture at the 84th passage. Myofibril-like thin filaments (arrow) were found, but Z-band like material could not be found. (The bar represents 1 μm × 12,800).

*Oncogene analysis*

High molecular weight DNA was purified from the cryopreserved primary tumour and RMS-YM cells at the 40th passage. DNAs digested by EcoRI and HindIII were electrophoresed in a 0.7% agarose gel. Southern blot analysis was performed as described previously (Southern, 1975). N-*myc* and N-*ras* probes were provided by Dr Taya (National Cancer Center, Tokyo, Japan) and Dr Shimizu (Kyushyu University, Fukuoka, Japan), respectively.

For the detection of mutated *ras* genes, PCR and differential oligonucleotide dot hybridisation were performed according to the published methods (Nagata *et al.*, 1990).

Figure 3 Immunohistochemical staining of the cultured cells; reactivity with antibodies specific for desmin a and human myoglobin b, and mouse sera (1:40 dilution) as a negative control c. (The bar represents 20 μm × 167).

### Results

### *The establishment and growth of the RMS-YM cell line and heterotransplantation into nude mice*

The RMS-YM cell line was established in tissue culture as described in the Materials and methods. The monolayer cultured cells were polygonal, round or spindle-shaped, and irregular in size. The cells grew in multilayers and formed many focal piles of cells. The growth rate gradually accelerated with subcultivation at early passages and finally became stable with a doubling time of about 30 h at the 30th, 42nd and 80th passages.

Cultured cells at the 12th and 80th passages were transplanted into two and three nude mice, respectively. Although tumour growth of the cells transplanted at the 12th passage was not noted on the back of one mouse, subcutaneous tumour nodules were noted on the backs of other four mice about 20 days after inoculation. These tumours then grew rapidly. We could not find gross tumour metastasis in any of these five mice.

### *Histopathological findings*

Hematoxylin-eosin stained sections of the RMS-YM cells, and the subcutaneous tumour in the nude mouse showed undifferentiated pleomorphic histological results similar to those of the patient tumour (Figure 1). Fine stippled granules positively stained by the PAS reaction were observed in the cytoplasm of most cells.

By electron microscopy, myofibril-like thin filaments were found in the RMS-YM cells (Figure 2) and heterotransplanted tumour, but dense Z-band like material was not found.

Figure 4 Immunoblot analysis of desmin; cell lysates of the cryopreserved primary tumour (Tumour), RMS-YM cells (Cell Line) and cultured human embryonal fibroblasts (Fibroblast). Anti-desmin antibody (Desmin) and mouse sera (1:40 dilution) as a negative control (NC) were used as the first antibody.

*Reactivity with antibodies specific for desmin and human myoglobin*

The RMS-YM cells were shown to be positive for desmin and human myoglobin by immunohistochemical staining (Figure 3). Furthermore, in immunoblot analysis using anti-desmin antibody, multiple thick bands of 45–50 kd were observed in the primary tumour. A clear single band of 50 kd, corresponding to the molecular weight of desmin, was detected in the RMS-YM cells (Figure 4). Some small molecules consisting of incomplete or immature desmin were thought to be present in the primary tumour, because the same pattern as shown in Figure 4 was recognised three times by immunoblot analysis. A faint band of 17.8 kd was also detected using anti-human myoglobin antibody in the primary tumour and in RMS-YM cells (data not shown).

*EIA of striated muscle system specific enzymes*

The determinations of β-enolase, CK-MM and CA-III by EIA are shown in Table I. High levels of β-enolase, CK-MM and CA-III were found in the primary tumour and in RMS-YM cells.

**Table I** Determination of β-enolase, CK-MM and CA-III

| | *β-enolase* | *CK-MM* *(ng mg$^{-1}$* | *CA-III* *soluble protein)* |
|---|---|---|---|
| *Cell line* | | | |
| RMS-YM | 508.9 | 31.9 | 25.9 |
| Fibroblast | 78.5 | 5.7 | 6.4 |
| *Tumour tissue* | | | |
| YM | 423.9 | 104.9 | 25.8 |
| rhabdomyosarcoma (mean: $n = 6$) | 77.2 | 67.7 | 33.0 |
| neuroblastoma (mean: n = 6) | 4.0 | 2.4 | 6.3 |
| Striated muscle | 15400.0 | 90800.0 | 20200.0 |

β-enolase: enolase β subunit; CK-MM: creatine kinase M subunit; CA-III: carbonic anhydrase III; Fibroblast: cultured human embryonal fibroblasts; YM: primary tumour of the patient.

*Chromosome analysis*

Karyotypic analysis was performed according to the international nomenclature system (ISCN 1985). Fifty two, 20 and 50 metaphases at the 25th, 35th and 77th passages, respectively, were photographed and karyotyped. The numbers of individual chromosomes varied from cell to cell and hyperdiploidy was common. The modal numbers were 54, 56 and 54 at the 25th, 35th and 77th passages, respectively.

A representative karyotype is shown in Figure 5. The most interesting finding was the presence of homogeneously staining regions (HSRs) on the long arm of chromosome 12 and on the short arm of chromosome 19, ins (12;hsr) (q15;hsr) and der (19) t (19;hsr;?) (19qter→19p13.1:: hsr :: ?), respectively. Unfortunately, however, it was not possible to perform chromosomal analyses on the primary tumour.

**Figure 5** A representative karyotype of RMS-YM cells at the 77th passage. Full description of the karyotype: 55, XY, −1, −2, −7, +8, −9, −10, −11, −16, −20, −22, +17 mars. Mars are as follows: M1: t (1q6p); M2: der (1) t (1;3) (p36;p21); M3: del (2) (q21); M4: t (2q8q); M5: t (7p16q); M6: der (7) t (7;7) (p15; q22); M7: del (7) (p11.2); M8: der (8) t (6;8) (q13;p21); M9: ins (12;hsr) (q15;hsr); M10: der (19) t (19;hsr;?) (19qter→19p13.1 :: hsr :: ?); M11: unidentified; M12: unidentified; M13: unidentified; M14: unidentified; M15: unidentified; M16: unidentified; M17: unidentified.

*Oncogene analysis*

We analysed N-*myc* and N-*ras* genes by Southern blotting, but N-*myc* and N-*ras* genes were not amplified or rearranged in the primary tumour or in RMS-YM cells (data not shown). The mutations at the 12th, 13th and 61st codons of Ki-*ras*, Ha-*ras* and N-*ras* genes were also analysed by the PCR method and dot hybridisation assays using synthetic oligonucleotide probes. No mutated *ras* genes were detected in the primary tumour or in RMS-YM cells (data not shown).

### Discussion

We have established a new human embryonal rhabdomyosarcoma cell line, RMS-YM, in tissue culture. As described in the Results, RMS-YM cells showed morphological features similar to those of the primary tumour. Myofibril-like thin filaments were found by electron microscopy. The expression of desmin and human myoglobin, and high levels of striated muscle system specific enzymes were also recognised. Tumourigenicity of the RMS-YM cells was shown by heterotransplantation into nude mice. These data indicate strongly that this cell line was derived from the primary rhabdomyosarcoma of the patient.

The most interesting cytogenetic features of the RMS-YM cell line are as follows: (1) the presence of HSRs at all passages examined in the form of ins (12;hsr) (q15;hsr) and der (19) t (19;hsr;?) (19qter→19p13.1 :: hsr :: ?), (2) structural rearrangement of the 3p21 region in the form of der (1) t (1;3) (p36;p21), as shown in Figure 5. The latter is supported by the fact that chromosome 3p14–21 has been reported as a site of nonrandom chromosomal change in rhabdomyosarcoma (Trent *et al.*, 1984).

The abnormality of chromosome 1 with a breakpoint at p11→p21 is a frequent finding in childhood malignancies (Douglass *et al.*, 1985). A specific chromosomal abnormality, t (2;13) has been observed in patients with very advanced disease in all types of rhabdomyosarcomas (Turc-Carel *et al.*, 1986; Douglass *et al.*, 1987), but these abnormalities were not found in RMS-YM cells.

HSR means amplification of a specific DNA fragment, which is found to occur in cellular adaptation to selective conditions, such as anti-cancer drug administration. The amplifications of dihydrofolate reductase gene and multidrug-resistance I gene are well known (Alt *et al.*, 1978; Roninson *et al.*, 1984). The RMS-YM cell line was established from a recurrent tumour, but it did not show any drug resistance when cultured in medium containing adriamycin (data not shown). Gene amplification is also suggested as a possible mechanism of tumourigenesis and tumour progression. Amplification of c-*myc* and N-*myc* has been reported in a variety of tumours, in which HSRs or double minutes were occasionally observed (Kohl *et al.*, 1983; Bishop, 1987). N-*myc* and N-*ras* genes were studied by Southern blotting, but the results showed only single-copy levels of the genes in the primary tumour and in RMS-YM cells. At present it is still unknown what genes are involved in HSRs of RMS-YM cells. Recently Roninson (1983) has developed a denaturation – renaturation gel technique for the detection of amplified sequences. If this technique can be employed to clone amplified DNA, new genes that affect the tumourigenesis of rhabdomyosarcoma may be identified.

As described in the Introduction, point mutations of Ki-*ras* or N-*ras* genes have been found in the RD human rhabdomyosarcoma cell line (Bos *et al.*, 1984; Chardin *et al.*, 1985), and tumours of human embryonal rhabdomyosarcoma (Stratton *et al.*, 1989). We also analysed mutations at the 12th, 13th and 61st codons of Ki-*ras*, Ha-*ras* and N-*ras* genes. However, no mutated *ras* genes were detected in the primary tumour or in RMS-YM cells.

RMS-YM cells may provide a system for the study of gene amplification in rhabdomyosarcoma. Further investigations are now in progress to determine the nature of amplified genes in the form of HSRs using this cell line.

We thank Ms Kyoko Sawai and Ms Kiyomi Inoue for technical assistance, and Ms Miki Funabashi for preparation of the manuscript.

### References

ALT, F.W., KELLEMS, R.E., BERTINO, J.R. & SCHIMKE, R.T. (1978). Selective multiplication of dihydrofolate reductase genes in methotrexate-resistant variants of cultured murine cells. *J. Biol. Chem.*, **253**, 1357.

BISHOP, J.M. (1987). The molecular genetics of cancer. *Science*, **235**, 305.

BOS, J.L., VERLAAN-DE VRIES, M., JANSEN, A.M., VEENEMAN, G.H., VAN BOOM, J.H. & VANCK EB, A.J. (1984). Three different mutations in codon 61 of the human N-*ras* gene detected by synthetic oligonucleotide hybridization. *Nucleic Acids Res.*, **12**, 9155.

CAVENEE, W.K., DRYJA, T.P., PHILLIPS, R.A. & 6 others (1983). Expression of recessive alleles by chromosomal mechanisms in retinoblastoma. *Nature*, **305**, 779.

CHARDIN, P., YERAMIAN, P., MADAULE, P. & TAVITIAN, A. (1985). N-*ras* gene activation in the RD human rhabdomyosarcoma cell line. *Int. J. Cancer*, **35**, 647.

CLAYTON, J., PINCOTT, J.R., VAN DEN BERGHE, J.A. & KEMSHEAD, J.T. (1986). Comparative studies between a new human rhabdomyosarcoma cell line, JR-1 and its tumour of origin. *Br. J. Cancer*, **54**, 83.

DOUGLASS, E.C., GREEN, A.A., HAYES, F.A., ETAUBANAS, E., HOROWITZ, M. & WILLIAMS, J.A. (1985). Chromosome 1 abnormalities: a common feature of pediatric solid tumors. *J. Natl Cancer Inst.*, **75**, 51.

DOUGLASS, E.C., VALENTINE, M., ETCUBANAS, E. & 4 others (1987). A specific chromosomal abnormality in rhabdomyosarcoma. *Cytogenet. Cell Genet.*, **45**, 148.

GARSON, J.A., CLAYTON, J., MCINTYRE, P. & KEMSHEAD, J.T. (1986). N-*myc* oncogene amplification in rhabdomyosarcoma at release. *Lancet*, **i**, 1496.

KATO, K., OKAGAWA, Y., SUZUKI, F., SHIMIZU, A., MOKUNO, K. & TAKAHASHI, Y. (1983). Immunoassay of human muscle enolase subunit in serum: a novel marker antigen for muscle diseases. *Clin. Chim. Acta*, **131**, 75.

KATO, K. & MOKUNO, K. (1984). Distribution of immunoreactive carbonic anhydrase III in various human tissues determined by a sensitive enzyme immunoassay method. *Clin. Chim. Acta.*, **141**, 169.

KATO, K. & SHIMIZU, A. (1986). High sensitive enzyme immunoassay for human creatine kinase MM and MB isozymes. *Clin. Chim. Acta.*, **158**, 99.

KOHL, N.E., KANDA, N., SCHRECK, R.R. & 4 others (1983). Transposition and amplification of oncogene-related sequences in human neuroblastomas. *Cell*, **35**, 359.

KOUFOS, A., HANSEN, M.F., LAMPKIN, B.C. & 4 others (1984). Loss of alleles at loci on human chromosome 11 during genesis of Wilms' tumour. *Nature*, **309**, 170.

KOUFOS, A., HANSEN, M.F., COPELAND, N.G., JENKINS, N.A., LAMPKIN, B.C. & CAVENEE, W.K. (1985). Loss of heterozygosity in three embryonal tumours suggests a common pathogenetic mechanism. *Nature*, **316**, 330.

MITANI, K., KUROSAWA, H., SUZUKI, A. & 7 others (1986). Amplification of N-*myc* in a rhabdomyosarcoma. *Jpn. J. Cancer Res.*, **77**, 1062.

NAGATA, Y., ABE, M., KOBAYASHI, K. & 5 others (1990). Glycine to aspartic acid mutations at codon 13 of c-Ki-*ras* gene in human gastro-intestinal cancers. *Cancer Res.*, **50**, 480.

NANNI, P., SCHIAFFINO, S., DE GIOVANNI, C. & 7 others (1986). RMZ: A new cell line from a human alveolar rhabdomyosarcoma. *In vitro* expression of embryonic myosin. *Br. J. Cancer*, **54**, 1009.

NAOE, T., NOZAKI, N., YAMADA, K. & 4 others (1989). Diversity of cellular molecules in human cells detected by monoclonal antibodies reactive with c-*myc* proteins produced in *Escherichia coli*. *Jpn. J. Cancer Res.*, **80**, 747.

ORKIN, S.H., GOLDMAN, D.S. & SALLAN, S.E. (1984). Development of homozygosity for chromosome 11p markers in Wilms' tumours. *Nature*, **309**, 172.

RONINSON, I.B. (1983). Detection and mapping of homologous, repeated and amplified DNA sequences by DNA renaturation in agarose gels. *Nucleic Acids Res.*, **11,** 5413.

RONINSON, I.B., ABELSON, H.T., HOUSMAN, D.E., HOWELL, N. & VARSHAVSKY, A. (1984). Amplification of specific DNA sequences correlates with multi-drug resistance in Chinese hamster cells. *Nature*, **309,** 626.

SEABRIGHT, M. (1971). A rapid banding technique for human chromosomes. *Lancet*, **ii,** 971.

SEKIGUCHI, M., SHIROKO, Y., SUZUKI, T., IMADA, M., MIYAHARA, M. & FUJII, G. (1985). Characterization of a human rhabdomyosarcoma cell strain in tissue culture. *Biomed. Pharmacother.*, **39,** 372.

SOUTHERN, E. (1975). Detection of specific sequence among DNA fragments separated by gel electrophoresis. *J. Mol. Biol.*, **98,** 503.

STRATTON, M.R., FISHER, C., GUSTERSON, B.A. & COOPER, C.S. (1989). Detection of point mutations in N-*ras* and K-*ras* genes of human embryonal rhabdomyosarcomas using oligonucleotide probes and the polymerase chain reaction. *Cancer Res.*, **49,** 6324.

SUTOW, W.W., FERNBACH, D.J. & VIETTI, T.J. (1984). *Clinical Pediatric Oncology* (3rd Edition). C.V. Mosby: St. Louis.

TRENT, J., CASPER, J., MELTZER, P., THOMPSON, F. & FOGH, J. (1985). Nonrandom chromosome alterations in rhabdomyosarcoma. *Cancer Genet. Cytogenet.*, **16,** 189.

TURC-CAREL, C., LIZARD-NACOL, S., JUSTRABO, E., FAVROT, M., PHILIP, T. & TABONE, E. (1986). Consistent chromosomal translocation in alveolar rhabdomyosarcoma. *Cancer Genet. Cytogenet.*, **19,** 361.